# Proceedings of the Second International Symposium for Semantic Mining in Biomedicine

**DOI:** 10.1186/1471-2105-7-S3-S1

**Published:** 2006-11-24

**Authors:** Sophia Ananiadou, Juliane Fluck

**Affiliations:** 1 School of Computer Science, National Centre for Text Mining, Manchester Interdisciplinary Biocentre, University of Manchester, Oxford Road, M13 9PL, Manchester, UK; 2 Fraunhofer Institute SCAI, Schloss Birlinghoven, 53754 St. Augustin, Germany

## Introduction

With an overwhelming amount of biomedical knowledge recorded in texts, it is not surprising that there is so much interest in techniques which can identify, extract, manage, integrate and exploit this knowledge, and moreover discover new, hidden or unsuspected knowledge. For this reason, in the past five years, there has been an upsurge of research papers and overviews [1-5] on the topic of text mining from biomedical literature. In order to facilitate knowledge discovery in biomedicine there is a need for approaches which harvest and integrate information from text, biological databases, ontologies and terminological resources. To discover knowledge hidden in the large amount of biomedical texts, we need text mining techniques which go to levels of linguistic processing deeper than simple lexical and syntactic processing. Text mining beyond surface levels requires semantic information. In order to carry out semantic mining certain prerequisites are assumed such as rich levels of linguistic and semantic annotation supported by ontologies and other knowledge sources that provide the semantics of the annotation. Semantic text mining enables us to capture the relevant content of documents according to user needs.

Semantic mining relies crucially on the following steps:

• named entity recognition

• discovery of semantic relations between entities

• event discovery

The current limitations of using existing terminological and ontological resources such as the Gene Ontology, Swiss-Prot, Entrez Gene, UMLS, and Mesh etc. have been well documented [6,7]. The entries are not useful for specific text searches and they do not contain the types of variability encountered in text. Results from the BioCreAtIvE [8] evaluation challenge reflect the problems related with named entity recognition in biomedicine. Term ambiguity (e.g. homologues, overlap with general language words) and term variation phenomena (spelling, morphological variants) account for the low performance of named entity recognisers for biology in comparison to newswire.

The existence of semantically annotated corpora for testing and training are of paramount importance for efficient semantic mining based on NLP techniques. The bio-text mining community must develop resources both in the form of semantically enriched lexical resources (from ontologies) or richly annotated corpora like GENIA [9], PennBioIE [10], GENETAG [11], etc.

Evaluation of information extraction systems (e.g. for protein-protein interactions) has been based till now on rather small benchmark sets mainly generated by research groups through use of their system. A promising path is presented with the BioCreative 06 [12] evaluation challenge which will provide common benchmark sets for training and testing of different systems for the extraction of protein-protein interactions out of full text articles. This assessment will show how close we are to providing solutions for real-world problems in molecular biology and biomedicine.

But this is just one part of the story so far: in order for text mining to exploit semantic data mining, we need to integrate the results of text mining not only with knowledge resources but most crucially with experimental data which will lead to biological discoveries. The analysis of high-throughput data in combination with textual extracted information about relationships of the investigated entities will allow biologists to make predictions about novel interactions.

## Semantic Mining in Biomedicine (SMBM) symposia series

The above-mentioned research problems have motivated the creation of a Network of Excellence – '*Semantic Interoperability and Data Mining in Biomedicine*' (Semantic Mining, ) – funded by the European Community since 2004 under the FP6 Programme 'Integrating and Strengthening the European Research Area'. The NoE has initiated the symposia series '*Semantic Mining in Biomedicine*' (SMBM) with a special focus on *content*-oriented methodologies and *semantic *resources – either controlled vocabularies, terminologies and formal domain ontologies, or conceptually as well as propositionally annotated corpora – in order to improve text-based biomedical knowledge management, e.g. through document classification, text or fact retrieval, information extraction, or (real) text mining. The second SMBM Symposium was organised by the Jena University Language and Information Engineering Laboratory. The aim of this symposium was to bring together the communities of molecular biology and genomics, chemo-informatics and pharma-informatics, text and data mining for biomedicine, medical informatics, and biological ontology design and engineering.

## Selected papers

Before turning to describe the content of each paper, we note that this supplement represents a selection of five papers from a set of papers and posters appearing in the proceedings of the conference [13]. The selected papers cover the following research areas: (1) adaptation of an information extraction approach to the biomedical domain, (2) inclusion of semantic knowledge for relation mining, (3) mining of disease gene relations, (4) propositional representations of biomedical knowledge and (5) data integration.

1. Lexical Adaptation of Link Grammar to the Biomedical Sublanguage: a Comparative Evaluation of Three Approaches by Sampo Pyysalo, Tapio Salakoski, Sophie Aubin and Adeline Nazarenko

This paper focuses on the evaluation of a link parser with respect to syntactic performance for two biomedical domains: transcription and interactions. Link grammar does not construct constituents in a tree-like hierarchy but builds simple relations between pairs of words. For the adaptation of the link grammar to the biomedical domain the authors review and analyze two lexical adaptation methods (lexicon expansion and morphological clues). Furthermore, they propose a third approach, which is based on the integration of a domain part of speech tagger and boosts the performance of their system.

2. An Environment for Relation Mining over Richly Annotated Corpora:The case of GENIA by Fabio Rinaldi, Gerold Schneider, Kaarel Kaljurand, Michael Hess and Martin Romacker

The authors present an environment which helps domain-experts to build deep-linguistic patterns for biomedical information extraction. The relation extraction system assumes the existence of named-entity annotation and the ontology (class hierarchy) of the entities as it is found in the GENIA corpus. The approach is based on a parser using a hand-written grammar combined with a statistical language model that calculates lexicalized attachment probabilities. It allows the user to maintain, edit and integrate syntactic patterns in an efficient way. The mapping from semantic patterns to syntactic patterns is implemented using a cascade of Prolog rules.

3. Recognizing mentions of fine-grained relations between prostate cancer and genes from Medline using machine learning techniques by Hong-Woo Chun Yoshimasa Tsuruoka, Jin-Dong Kim, Rie Shiba, Naoki Nagata, Teruyoshi Hishiki and Jun'ichi Tsujii

The authors recognised automatically relations between prostate cancer and gene terms with the ID tags of public biomedical databases. Using a maximum entropy-based named entity recognizer and a manually annotated corpus of gene and prostate cancer relations, they have also classified them into six categories that can be used to analyze the type of prostate cancers, genes, and their relations. The performance of the relation recogniser was boosted by including named entity recognition and that of the topic based relation recognition by the use of a combination of machine learning techniques and a manually annotated corpus.

4. A critical review of PASBio's argument structures for biomedical verbs by K. Bretonnel Cohen and Lawrence Hunter

The authors quantitatively evaluate the PASBio project whose task was to define a set of propositions and argument structures, linked to biomedically relevant verbs. Propositional representation schemes specify the particular types of relationships along with the number and type of related entities. PASBio's importance for information extraction is particularly relevant because of the high arity of its propositions and the individual thematic roles. A comparison between Levin's verbs classes with PASBio's is provided.

5. Data Integration through data Elements: Mapping Data Elements to Terminological Resources by Fleur Mougin, Anita Burgun and Olivier Bodenreider

The integration of data elements from heterogeneous knowledge bases is a bottleneck in their use and in the dissemination of the knowledge. This paper discusses how to map data elements to terminological resources, such as the UMLS and NCI caDSR, in order to automatically integrate data across various systems and databases. The authors proposed direct, indirect and heuristic mapping approaches and evaluated their performance. The direct mapping approach is straightforward and reasonable, while it is limited to mapping the database entries to lexically similar entries. The indirect mapping approach makes use of the values associated with the database entries to do the mapping. In addition, the heuristic mapping approach handles some specific mapping instances.
